# Neuroprotection by taurine in ethanol-induced apoptosis in the developing cerebellum

**DOI:** 10.1186/1423-0127-17-S1-S12

**Published:** 2010-08-24

**Authors:** Andrey G Taranukhin, Elena Y Taranukhina, Pirjo Saransaari, Irina M Podkletnova, Markku Pelto-Huikko, Simo S Oja

**Affiliations:** 1 Brain Research Center, University of Tampere Medical School, Tampere, Finland; 2 Laboratory of Comparative Somnology and Neuroendocrinology, Sechenov Institute of Evolutionary Physiology and Biochemistry, St.-Petersburg, Russia; 3 Department of Developmental Biology, University of Tampere Medical School and Department of Pathology, Tampere University Hospital, Tampere, Finland; 4 Department of Paediatrics, Tampere University Hospital, Tampere, Finland

## Abstract

**Background:**

Acute ethanol administration leads to massive apoptotic neurodegeneration in the developing central nervous system. We studied whether taurine is neuroprotective in ethanol-induced apoptosis in the mouse cerebellum during the postnatal period.

**Methods:**

The mice were divided into three groups: ethanol-treated, ethanol+taurine-treated and controls. Ethanol (20% solution) was administered subcutaneously at a total dose of 5 g/kg (2.5 g/kg at time 1 h and 2.5 g/kg at 3 h) to the ethanol and ethanol+taurine groups. The ethanol+taurine group also received two injections of taurine (1 g/kg each, at time zero and at 4 h). To estimate apoptosis, immunostaining for activated caspase-3 and TUNEL staining were made in the mid-sagittal sections containing lobules I-X of the cerebellar vermis at 12 or 8 hours after the first taurine injection. Changes in the blood taurine level were monitored at each hour by reverse-phase high-performance liquid chromatography (HPLC).

**Results:**

Ethanol administration induced apoptosis of Purkinje cells on P4 in all cerebellar lobules, most extensively in lobules IX and X, and on P7 increased the number of activated caspase-3-immunoreactive and TUNEL-positive cells in the internal layer of the cerebellum. Administration of taurine significantly decreased the number of activated caspase-3-immunoreactive and TUNEL-positive cells in the internal layer of the cerebellum on P7, but had no effect on Purkinje cells in P4 mice. The high initial taurine concentration in blood of the ethanol+taurine group diminished dramatically during the experiment, not being different at 13 h from that in the controls.

**Conclusions:**

We conclude that the neuroprotective action of taurine is not straightforward and seems to be different in different types of neurons and/or requires prolonged maintenance of the high taurine concentration in blood plasma.

## Background

A moderate alcohol intake may not be harmful and has even beneficial effects in prevention of cardiovascular diseases, for example [1]. On the other hand, heavy alcohol consumption is associated with the reduced brain mass, neuronal loss, neuropathological changes and results in the impairment of cognitive functions, amnesia, dementia and even a significant increase in mortality [2-4]. In adult rats a short-term increase in the blood ethanol concentration up to 6 g/l has not been toxic to the central nervous system [5]. However, in the developing nervous system the situation is quite different. The blood ethanol concentration above 0.5 g/l in mice during their early postnatal life induces mild apoptotic neurodegeneration [6], which becomes dose-dependently more severe from the concentration of 2 g/l upward [7].Intrauterine exposure of the human fetus to ethanol due heavy drinking or repeated binge drinking of pregnant women causes a wide spectrum of developmental disorders known as the fetal alcohol syndrome [8,9]. The human fetal brain is particularly sensitive to the adverse effects of alcohol during the last trimester of pregnancy, the period of synaptogenesis, also known as the brain growth spurt period. In rodents, the same period of increased sensibility to ethanol is during the early postnatal period [10]. It has been well documented that acute ethanol exposure to neonatal mice induces neuronal loss by apoptosis [6,7,11-14]. Prevention of ethanol-induced apoptosis can save a huge amount of neurons and significantly decrease the harmful consequences of alcohol intoxication.

Taurine is a simple sulphur-containing free amino acid abounding in electrically excitable tissues such as brain, retina, heart and skeletal muscles [15]. It is involved in a wide range of physiological processes, for example, in osmoregulation, lipid metabolism, intracellular calcium regulation, neuronal development, neuromodulation and cell protection [16-20]. Recent findings also imply taurine in apoptosis regulation [21-24]. In the present work we focused on the possible protection of Purkinje cells and neurons in the internal layer of the developing cerebellum against apoptosis induced by acute ethanol administration. Among possible means to prevent pathological apoptosis taurine is very attractive since it is a naturally-occurring and non-toxic compound.

## Methods

### Animals and treatments

Adult NMRI mice for breeding were purchased from Harlan, Netherlands. Four (P4) - and seven (P7) - day old infant male mice were used in the experiments (day of birth is day 0). The experiments on animals were carried out in accordance with the European Community Council Directive 86/609/EEC. All efforts were made to reduce their number and suffering. The mice in each litter were divided into three groups: ethanol-treated, ethanol+taurine-treated and controls. To induce acute alcohol intoxication ethanol was mixed in sterile saline to a 20 % solution and administered subcutaneously at a total dose of 5 g/kg (2.5 g/kg at time 1 h and 2.5 g/kg again at 3 h) to the ethanol and ethanol+taurine groups. This dosing regimen was well documented to produce an elevation in the blood alcohol concentration above 2 g/l for at least 8 hours and led to significant and widespread apoptotic neurodegeneration in the developing brain [7] and cerebellum [25]. The ethanol+taurine group also received two injections of taurine (1 g/kg diluted with saline). The first taurine injection was given one hour before the first ethanol injection and the second one hour after the second ethanol injection. The control animals were given saline subcutaneously. Twelve (P4) and eight hours (P7) after the first ethanol injection the mice were killed by decapitation. Blood samples from each animal were collected separately in lithium-heparin tubes and centrifuged at 1750 rpm for 10 min to obtain plasma. The samples were frozen until HPLC analyses. The cerebella were rapidly excised and fixed in 4 % paraformaldehyde in phosphate buffered saline for at least 3 days at 4^o^ C. They were after the routine histological processing embedded in paraffin and cut with a microtome into 5-μm thick mid-sagittal sections containing lobules I-X of the cerebellar vermis.

### High Performance Liquid Chromatography (HPLC)

The concentration of taurine in the blood serum was measured using HPLC with fluorescent detection after precolumn derivatization with o-phthaldialdehyde (OPA) using the analysis equipment system of Shimadzu Scientific Instruments (Kyoto, Japan). The separation column was 4.6 x 250 mm Ultropac 8 Resin, lithium form (Farmacia, Denmark). Derivatization of taurine was performed with the OPA reagent (0.2 % OPA, 0.1 % mercaptoethanol and 1 % ethanol in 1 M borate buffer, pH 10.4). The elution was done with lithium citrate buffers in the following order: (1) 0.2 M, pH 2.80, (2) 0.3 M, pH 3.00, (3) 0.5 M, pH 3.15, (4) 0.9 M, pH 3.50, and (5) 1.6 M, pH 3.30. Fluorescence of taurine derivatives was measured with an RF-10A detector using the excitation and emission wavelengths set at 340 nm and 450 nm, respectively. The concentrations of taurine were finally estimated using a commercial amino acid mixture (Pickering, UK) as an external standard and diamino-n-butyrate as an internal standard.

### Immunohistochemistry

The sections were deparaffinized with xylene and hydrated in a graded ethanol series to distilled water. After antigen retrieval by microwave [20 min at 1000 W in 0.01 M citrate buffer (pH 6.0)], washing in phosphate buffered saline and blocking with 0.5 % hydrogen peroxide in this buffer for 20 min, the specimens were preincubated for 30 min in serum-blocking solution (1 % bovine serum albumin and 0.3 % Triton X-100 in the above buffer). The specimens were thereafter incubated with polyclonal activated caspase-3 antibody [cleaved caspase-3 (Asp 175) antibody, Cell Signaling Technology Inc., diluted 1:200 in serum-blocking solution] in moist chambers overnight at 4°С. After incubation with the primary antibody, the sections were incubated with biotinylated secondary antibody (goat anti-rabbit 1:500 in blocking solution) and ABC complex (Vectastain Elite ABC Kit, Vector Laboratories, Inc.), each for 30 min. Diaminobenzidine was used as a chromogen to visualize the sites expressing activated caspase-3 immunoreactivity. The sections for negative control were incubated without the primary antibodies to rule out nonspecific staining. Finally, the sections were counterstained by hematoxylin-eosin to better reveal histological details, dehydrated and mounted.

### Detection of cell death in situ

DNA fragmentation is one of the most precise markers by which apoptotic cells are recognized. In order to detect DNA fragmentation of cell nuclei, terminal deoxynucleotidyl transferase-mediated dUTP nick end-labeling (TUNEL) reaction was applied to the paraffin sections using the In Situ Cell Death Detection Kit, POD (Roche Applied Science, Germany). After deparaffinization, the sections were irradiated with microwaves in 0.01 M citric acid buffer (pH 6) for 10 min at 750 W. No inhibition of endogenous peroxidase was performed because H_2_O_2_ weakens terminal deoxynucleotidyl transferase activity [26] and induces DNA breaks [27]. Sections were incubated with the TUNEL reaction mixture for 60 min at 37°С. Further incubation with peroxidase-conjugated antibody was performed for 30 min at 37°С. The sections were stained with diaminobenzidine for 10 min at the room temperature and then counterstained with hematoxylin-eosin.

### Image analysis and cell counting

The sections were processed in parallel under standardized conditions for immunostaining or TUNEL to minimize variability in labeling conditions. An image analysis system comprising of an IBM PC, Nikon Microphot-FXA microscope, SensiCam digital camera (PCO Computer Optics GmbH), Image-Pro Plus (Media Cybernetics) was used for analysis of caspase-3 immunoreactivity and TUNEL-staining in the histological sections of the cerebellum. At least five sections cut at the same level of the cerebellar vermis from every animal were analyzed. The amount of cells labeled for active caspase-3 or TUNEL were calculated in every slice in each lobule and the area of each lobule was also measured. The data are presented as the average number of labelled cells per mm^2^ for each experimental group.

### Statistical analysis

One-way analysis of variance (ANOVA) was used to compare the number of activated caspase-3-immunoreactive cells and TUNEL-positive cells among the experimental groups. When ANOVA showed a significant difference, the post hoc Bonferroni test was applied to demonstrate the difference. Each value is expressed as mean ± standard deviation. Differences were considered significant when the calculated *p* value was < 0.05.

## Results

In the present work we focused on the Purkinje cells and neurons in the internal granular cell layer of the cerebellum. The Purkinje cells were identified by their size, shape and specific localization in the cerebellar lobules. Because we did not make specific labeling to identify different types of neurons and glial cells in the internal granular cell layer of the cerebellum, we refer to these cells as internal granular layer (IGL) cells. We studied the possible protective effect of taurine against ethanol-induced apoptosis in the Purkinje cells on P4 and the IGL cells on P7 mice because of the extremely high sensitivity of these cell types at these ages to ethanol-induced apoptotic neurodegeneration.

### Effects of taurine on ethanol-induced caspase-3 activation

**Figure 1:**
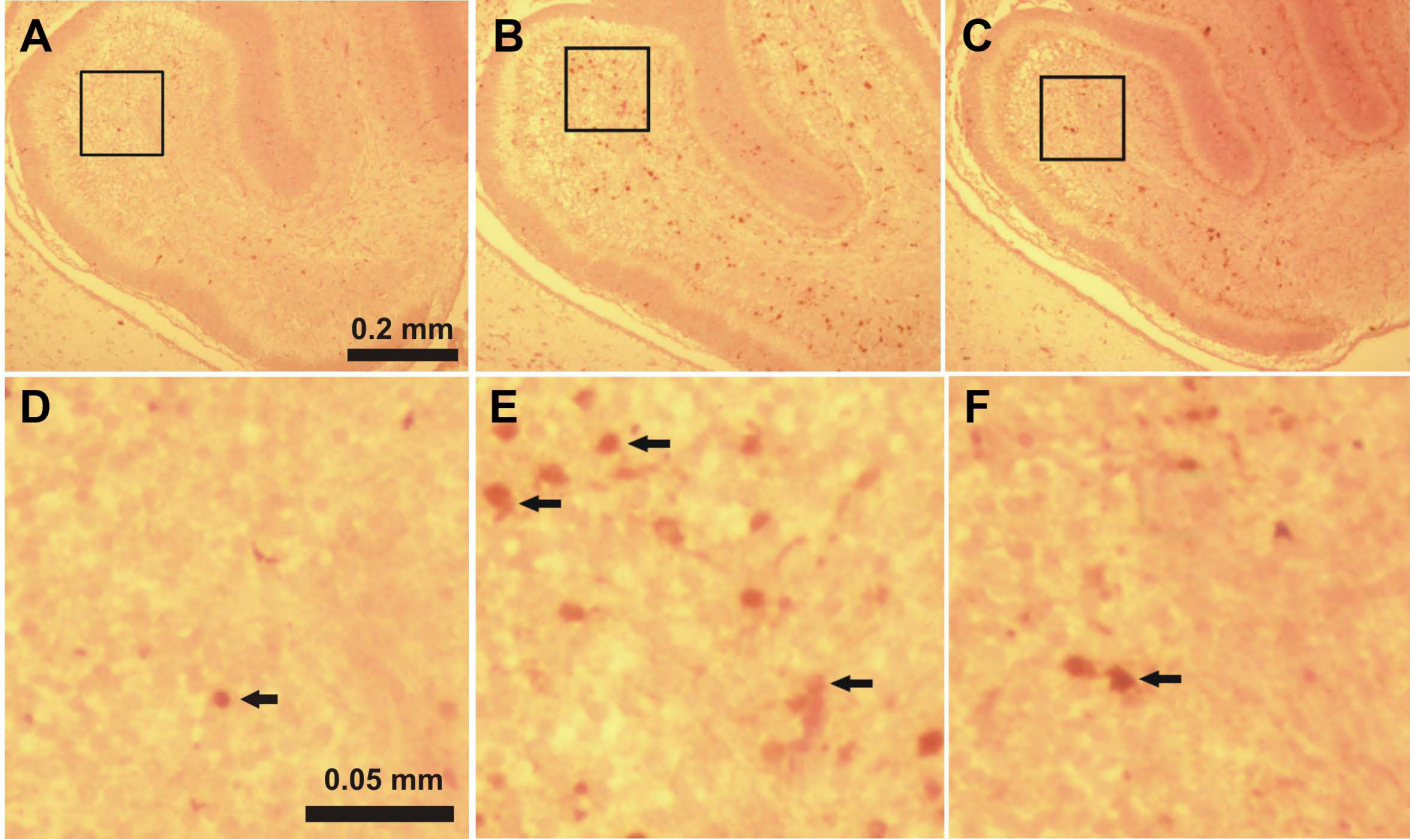
**Immunostaining for activated caspase-3 in lobule II of the cerebellum of 7-day-old mice 8 hours after the first ethanol injection** A, D: control group, B, E: ethanol-treated group, C, F: ethanol+taurine-treated group. The activated caspase-3-immunoreactive cells in the IGL are indicated by black arrows.

**Figure 2:**
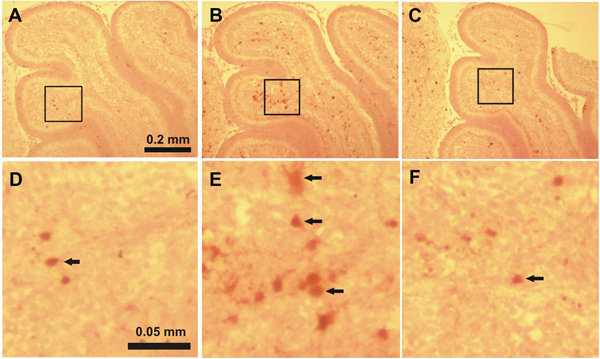
**Immunostaining for activated caspase-3 in lobule IV-V of the cerebellum of 7-day-old mice 8 hours after the first ethanol injection** A, D: control group, B, E: ethanol-treated group, C, F: ethanol+taurine-treated group. The activated caspase-3-immunoreactive cells in the IGL are indicated by black arrows.

**Figure 3:**
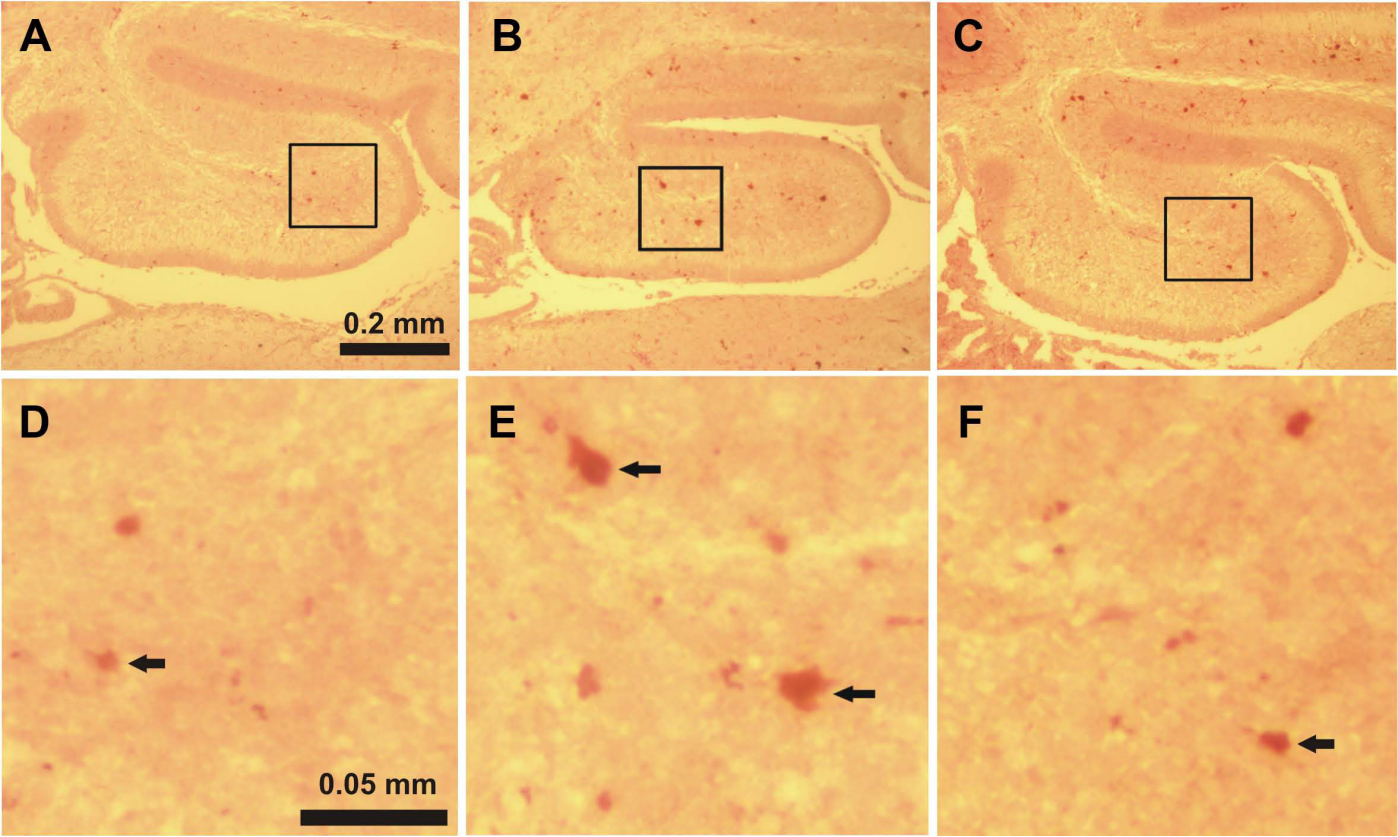
**Immunostaining for activated caspase-3 in lobule X of the cerebellum of 7-day-old mice 8 hours after the first ethanol injection** A, D: control group, B, E: ethanol-treated group, C, F: ethanol+taurine-treated group. The activated caspase-3-immunoreactive cells in the IGL are indicated by black arrows.

Activated caspase-3-immunoreactive (IR) cells in the IGL found in each cerebellar lobule in the saline-treated control mice on P7 evidences physiological cell death which normally occurs at this period of development (Figure 1A, 1D; Figure 2A, 2D; Figure 3A, 3D). Only a few activated caspase-3-IR Purkinje cells were found in the saline-treated control mice on P4.

**Figure 4:**
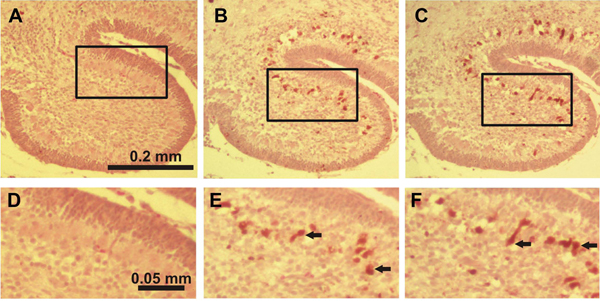
**Immunostaining for activated caspase-3 in lobule X of the cerebellum of 4-day-old mice 12 hours after the first ethanol injection** A, D: control group, B, E: ethanol-treated group, C, F: ethanol+taurine-treated group. The activated caspase-3-immunoreactive Purkinje cells are indicated by black arrows.

**Figure 5:**
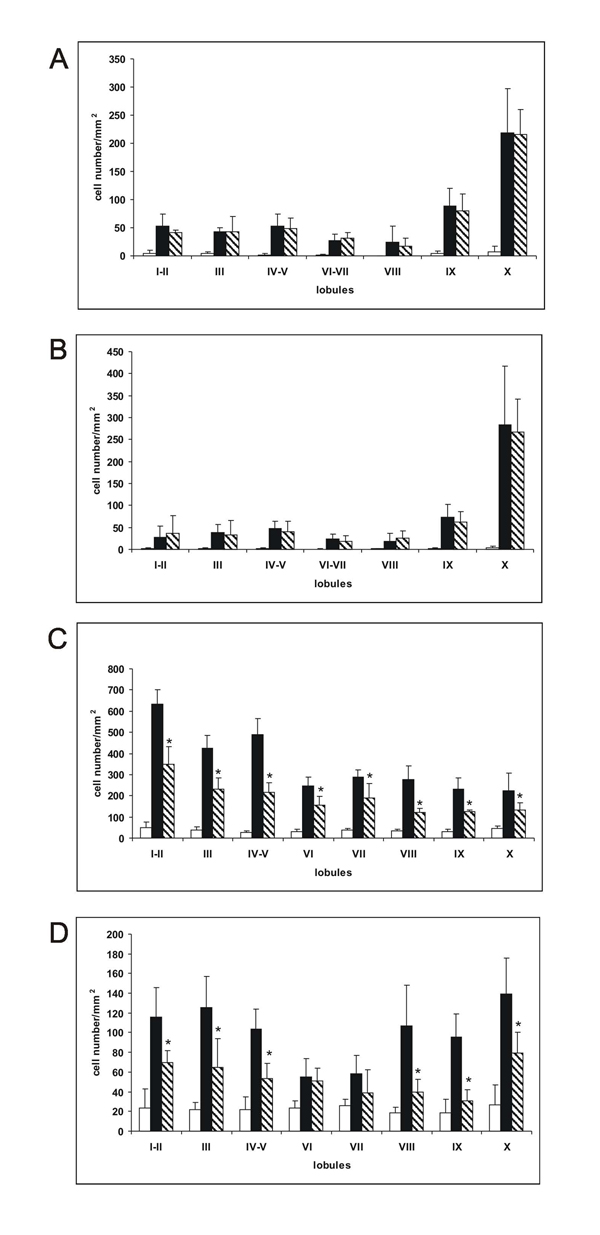
**Effects of taurine on ethanol-induced apoptosis in the developing cerebellum** (A) Number of activated caspase-3 immunoreactive Purkinje cells in the cerebellar lobules of 4-day-old mice in the control (open bars), ethanol-treated (filled bars) and ethanol+taurine-treated (hatched bars) mice. The results are given per mm^2^ with standard deviations. Number of animals in each group is 5. Ethanol significantly (P<0.01) increased the number of caspase-3 immunoreactive cells in all lobules. There were no significant differences between the ethanol and ethanol+taurine groups. (B) Number of apoptotic Purkinje cells (labeled by TUNEL assay) in the cerebellar lobules of 4-day-old mice in the control (open bars), ethanol-treated (filled bars) and ethanol+taurine-treated (hatched bars) mice. The results are given per mm^2^ with standard deviations. Number of animals in each group is 5. Ethanol significantly (P<0.01) increased the number of TUNEL-positive Purkinje cells in all lobules. There were no significant differences between the ethanol and ethanol+taurine groups. (C) Number of activated caspase-3-immunoreactive cells in the IGL in the cerebellar lobules of 7-day-old mice in the control (open bars), ethanol-treated (filled bars) and ethanol+taurine-treated (hatched bars) mice. The results are given per mm^2^ with standard deviations. Number of animals in each group is 5. Ethanol significantly (P<0.01) increased the number of caspase-3 immunoreactive cells in all lobules. The significance of differences between the ethanol and ethanol+taurine groups: *P<0.05.(D) The number of apoptotic cells (labeled by TUNEL assay) in the IGL in the cerebellar lobules of 7-day-old mice in the control (open bars), ethanol-treated (filled bars) and ethanol+taurine-treated (hatched bars) mice. The results are given per mm^2^ with standard deviation. Number of animals in each group is 5. Ethanol significantly (P<0.01) increased the number of TUNEL-positive cells in all lobules. The significance of differences between the ethanol and ethanol+taurine groups: *P<0.05.

In the ethanol-treated pups on P4 a large number of activated caspase-3-IR Purkinje cells were discernible in all lobules with the highest amount in lobules IX and X (Figure 4B, 4E). In lobules I-II, III, IV-V, VI-VII and VIII the number of capase-3-IR Purkinje cells was also significantly increased when compared to the control group (Figure 5A). On P7 ethanol administration induced very little response to caspase-3 activation in the Purkinje cell layer which hardly differed from the control group. However, ethanol treatment on P7 induced widespread activation of caspase-3 in the IGL (Figure 1B, 1E; Figure 2B, 2E; Figure 3B, 3E). The amount of activated caspase-3-IR IGL cells in each lobule was markedly greater than in the saline-treated pups (Figure 5C). The highest immunoreactivity for activated caspase-3 was found in I-II, III and IV-V vermian lobules.

In the ethanol+taurine-treated group on P4 the number of activated caspase-3-IR Purkinje cells in each lobule was approximately the same as in the pups treated only with ethanol and significantly higher than in the saline-treated control group (Figure 4; Figure 5A). However, in contrast to the Purkinje cells at age P4, taurine treatment significantly decreased the number of activated caspase-3-IR cells in the IGL on P7 in each lobule (Figure 1C, 1F; Figure 2C, 2F; Figure 3C, 3F; Figure 5C). The amount of rescued cells from caspase-3 activation were different in different lobules and varied from 34 % to 41 % for lobules VI, VII and X, and from 45 % to 57 % for lobules I-II, III, IV-V, VIII and IX.

### Effects of taurine on ethanol-induced DNA fragmentation

A few TUNEL-positive cells in the Purkinje cell layer on P4 and in the IGL on P7 were detected in the saline-treated control groups showing physiological cell death during normal development. The amount of cells undergoing physiological cell death in the Purkinje layer seems to be less than in the IGL and it was in accordance with our data on activated caspase-3 immunoreactivity.

**Figure 6:**
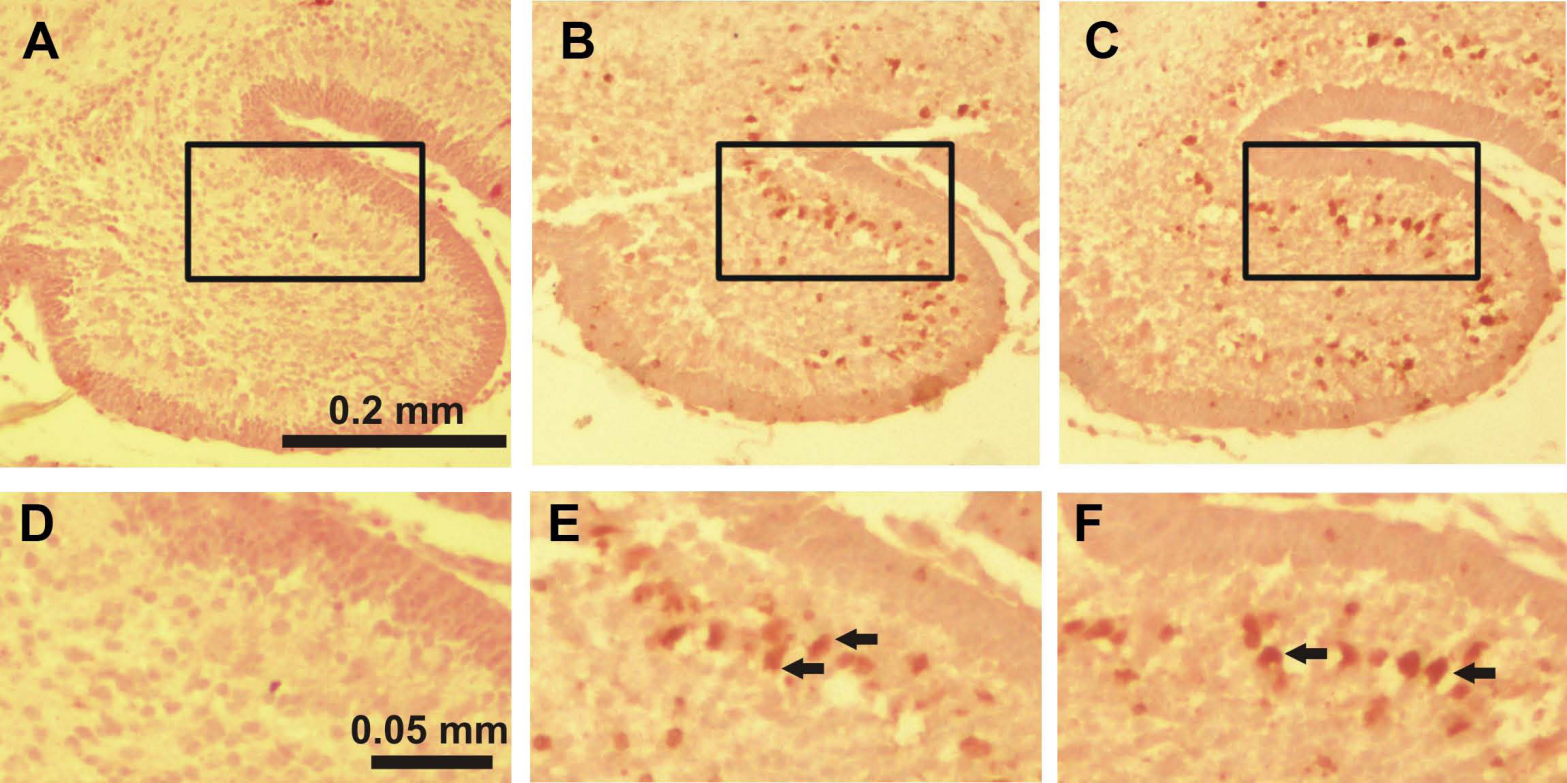
**TUNEL staining in lobule X of the cerebellum of 4-day-old mice 12 hours after the first ethanol injection** A, D: control group, B, E: ethanol-treated group, C, F: ethanol+taurine-treated group. TUNEL-positive Purkinje cells are indicated by black arrows.

Ethanol treatment significantly increased the number of cells with fragmented DNA labeled by the TUNEL assay in the Purkinje cell layer of P4 mice (Figure 5B). The largest number of TUNEL-positive Purkinje cells was found in lobules IX and X (Figure 6B, 6E), although in the other lobules studied the amount of TUNEL-positive Purkinje cells was also much greater than in the control group. At age P7, ethanol administration induced massive apoptosis in the IGL in all vermian lobules as indicated by the increased amount of TUNEL-positive cells (Figure 5D).

Taurine treatment did not change the amount of TUNEL-positive Purkinje cells on P4 in any lobule studied when compared to the pups treated only with ethanol (Figure 5B; Figure 6). The amount of TUNEL-positive Purkinje cells remained much greater than in the saline-treated control group. In the ethanol+taurine group at age P7 the number of TUNEL-positive IGL cells was reduced in comparison to the ethanol group, the change being statistically significant in all other lobules except lobules VI and VII (Figure 5D). Although taurine treatment decreased the number of TUNEL-positive IGL cells induced by acute alcohol administration, the number of TUNEL-positive cells in the ethanol+taurine group remained much larger than in the control group. Taurine preservation of IGL cells from DNA fragmentation varied in the lobules from 40 % (lobule I-II) to 68 % (lobule IX).

### Dynamics of taurine blood concentration changes during the experiments

**Figure 7:**
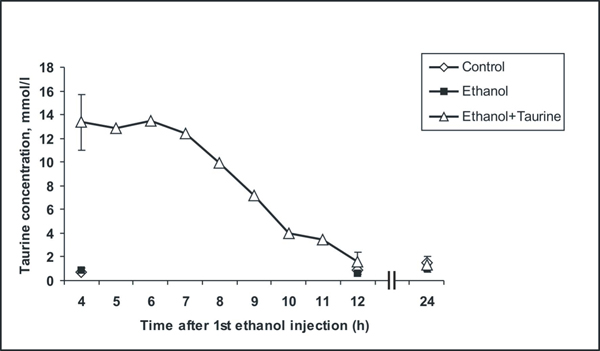
**Changes in the taurine concentration in blood plasma during the experiment in 7-day-old mice** Data are presented as mean values. Standard deviations are shown only for samples at 4 h, 12 h and 24 h after the first ethanol injection.

We measured the taurine concentration in blood serum during the experiments from 4 h onwards after the first ethanol injection (1 hour after last taurine injection) at every hour for 12 h and then again at 24 h (Figure 7). In the saline-treated control group and in the ethanol group of P7 mice the taurine concentration remained the same at 4 h, 12 h and 24 h. It was 1.00 ± 0.42 mmol/l in the control group and 0.85 ± 0.24 mmol/l in the ethanol group. Two subcutaneous injections of taurine each at the dose 1 g/kg (one at 0 h and second at 4 h) markedly increased the taurine level up to 13.36 ± 2.73 mmol/l at 4 h after the first ethanol injection. For four hours (4 h-7 h) the concentration in the blood was maintained at about the same high level of 13.20 ± 1.83 mmol/l and then it started to decline gradually and almost reached the control level by 12 h (1.59 ± 0.83 mmol/l).

## Discussion

The central nervous system is extremely sensitive to alcohol during development and the periods of vulnerability are temporally well defined. The time frames of vulnerability are different for different neuronal populations. In the developing mouse cerebellum acute alcohol intoxication thus induces massive neuronal death of Purkinje cells on P2-P6 and of cells in the IGL on P7-P9 [25]. In our experiments P4 and P7 mice pups were used to induce ethanol-induced degeneration of Purkinje and IGL cells, respectively. It is well established that alcohol induces neuronal death in the developing brain by apoptosis [11]. Apoptosis is a type of programmed cell death with specific morphologic features [28]. Immature neurons dying due to ethanol exposure, exhibit biochemical and ultrastructural features of apoptosis such as activation of caspase-3 [12,13], internucleosomal DNA fragmentation [7,14], clumping of nuclear chromatin, formation of spherical chromatin masses and nuclear membrane fragmentation [7,25,29].

Two major apoptotic pathways have been established: the death-receptor-mediated and the mitochondrial-mediated apoptosis [28], also known as the “extrinsic” and as “intrinsic” apoptotic pathways, respectively [30]. In both pathways, the caspases, a family of cysteine-dependent aspartate-directed proteases, play an important role in initiation, signal transduction and execution of apoptosis [31,32]. The extrinsic pathway is triggered by activation of death receptors localized at the cell membrane surface and it induces caspase-8 processing. The activated caspase-8 can directly or indirectly activate effector caspases such as caspases-3, -6 and -7. In the intrinsic pathway, many factors such as nitric oxide, oxidants and proapoptotic proteins, e.g. Bax, increase mitochondrial membrane permeability and release cytochrome C into the cytoplasm. Cytochrome C binds to Apaf-1 and procaspase-9, forming an apoptosome, leading to caspase-9 activation [33]. The active caspase-9 cleaves and activates effector caspases, including caspase-3. The activated effector caspases cleave many structural and functional proteins and activate DNase which destroys chromosomes and leads to cell death [31,32]. As shown with Bax-knock-out mice, ethanol-induced apoptosis in the developing brain is a Bax-dependent process which requires translocation of the Bax protein from the cytosol to mitochondria, disruption of mitochondrial membranes, release of cytochrome C and activation of caspase-3. All these events and also the absence of capase-8 activation indicate that alcohol induces apoptosis in the developing brain via intrinsic (mitochondrial-mediated) apoptotic pathways [34,35] .

In the present experiments we demonstrate that acute ethanol administration to P4 mice induces activation of caspase-3 and DNA fragmentation in Purkinje neurons in all vermian lobules studied. This finding is in concert with the results of other authors working with mice [25] and rats [36]. Ethanol administration to mice pups at age P7 markedly enhanced the number of activated caspase-3-IR and TUNEL-positive cells in the IGL but did not markedly affect the Purkinje cells. This time difference in alcohol sensitivity of different types of neurons is not surprising, being already shown in other studies [25].

The presence of taurine at high concentrations during the early ontogenesis is essential for normal development [37]. Taurine has been tested in treatment of many diseases, including cardiovascular disorders, epilepsy, macular degeneration, hepatic disorders, cystic fibrosis, Alzheimer’s disease and alcoholism [38]. It also interacts with the effects of ethanol [39]. For instance, it modulates ethanol-stimulated locomotion [40] and prolongs ethanol-induced sedation when given intracerebroventricularly to mice [41,42]. Furthermore, ethanol administration elicits an increase in extracellular taurine in the rat cerebral cortex and hippocampus [43]. However, many findings on taurine and ethanol interactions have been contradictory. For instance, in behavioural studies taurine pretreatment has reduced the duration of ethanol-induced sleep-time and attenuated the loss of righting reflex [44,45], not altered the ethanol-induced loss of righting reflex [46] or even enhanced it [41]. It seems that interactions of taurine and ethanol in the brain depend largely on the experimental set-up and the doses of ethanol and taurine administered [19].

Mounting recent evidence indicates that taurine is involved in apoptosis regulation and protects many cell types under different pathological conditions such as ischemia [22,23], high glucose level [47], oxidative stress [48], and ethanol intoxication [49]. In adult rats intraperitoneal taurine injections markedly increase the taurine concentration in blood plasma and brain microdialysates in a dose-dependent manner [50]. After a single injection of 1 g/kg taurine the maximal plasma and brain concentrations are reached within 20 min and they remain significantly higher than in controls for 3.5-4 h. The present two taurine injections at the dose of 1 g/kg each with the 4-h interval keep the level of taurine high for a more prolonged time. The present finding that in this manner taurine attenuated apoptosis in the IGL is in accordance with our previous study [49].

However, in P4 mice we got unexpected results that taurine had no effect on the ethanol-induced activation of caspase-3 and apoptosis in Purkinje cells. Taurine can be involved in apoptosis regulation by several ways. First of all, taurine decreases intracellular free Ca^2+^ by inhibiting all types of voltage-gated calcium channels and the N-methyl-D-aspartate (NMDA) receptor-gated calcium channel [24,51] and by increasing Ca^2+^ buffering in mitochondria [52]. It prevents in this manner activation of calpain (calcium-dependent protease) and protects mitochondrial membranes from disruption [24]. Taurine can also protect cells acting as antioxidants [15], scavenging at its different physiological concentrations many reactive oxygen and nitrogen species [53], although ethanol administration to neonatal rats may not induce oxidative stress to cerebellar granular neurons [54]. For cell survival a balance between the proapoptotic protein Bax and the antiapoptotic protein Bcl-2 is very important. A decrease in the Bcl-2 level in cells leads to translocation of Bax to mitochondria, disruption of their membranes and a release of cytohrome C from mitochondria to the cytosol [55]. Taurine application can restore the pool of Bcl-2 and protect cells against apoptosis [24]. Since ethanol-induced apoptosis is a Bax-dependent process [34], we suggest that restoration of the Bcl-2 level was one possible mechanism of apoptosis prevention in our experiments. Taurine may also be able to rescue cells from apoptosis after the release of cytochrome C from mitochondria. In ischemic cardiomyocytes taurine suppresses the formation of the Apaf-1/caspase-9 apoptosome, prevents caspase-9 activation and thereby preserves cells from apoptosis [22]. All these mechanisms may explain why taurine protects IGL neurons from ethanol-induced apoptosis in P7 mice.

It also seems important to consider another possibility of taurine protection against the adverse effect of ethanol, which is not directly related to apoptosis. Alcohol alters physical properties of membrane lipids, changing membrane fluidity and affecting physiologically important membrane enzymes, for instance, Na^+^/K^+^-ATPase activity [56-59]. The effects of alcohol on biological membranes depend on the duration of its administration. Acute ethanol administration increases the membrane fluidity [60-62], whereas chronic alcohol intake decreases the fluidity of membranes [60,63,64], which become more tolerant to the disordering effect of ethanol [60,63]. Acute ethanol exposure also decreases Na^+^/K^+^-ATPase activity in the cerebral cortex and brain stem [65] and kidneys of adult rats [66].

The experimental data on the chronic alcohol effect on Na^+^/K^+^-ATPase activity seem more contradictory. For instance, chronic alcohol intake has enhanced Na^+^/K^+^-ATPase activity in rat erythrocyte membranes [58] and in the brain [67]. However, in other studies the activity of Na^+^/K^+^-ATPase was significantly decreased after chronic alcohol consumption in human erythrocyte membranes [59] and in the brain and cerebellum of rat offspring upon chronic alcohol exposure in utero [68]. Taurine also acts as a membrane stabilizer and restores the depletion of Na^+^/K^+^-ATPase activity due to ozone exposure and prevents the depletion of the enzyme activity due to cholesterol enrichment [69]. Furthermore, taurine decreases the fluidity of biological membranes when coadministrated with calcium [70]. It seems thus possible that taurine in addition to its anti-apoptotic actions can reduce the adverse effects of acute ethanol administration acting as membrane stabilizer by the decreasing membrane fluidity and restoring the Na^+^/K^+^-ATPase activity.

Hovewer, the above considerations do not explain why taurine did not affect apoptosis of Purkinje cells on P4. An explanation may be the different functional properties of the neurons studied. The granular neurons in the IGL are glutamatergic and the Purkinje cells GABAergic. Ethanol can induce apoptosis in the developing brain by dual mechanisms, by either blocking NMDA receptors and or by excessively activating GABA_A_ receptors [7]. Taurine itself is an inhibitory amino acid which mimics GABA actions [71,72]. On the other hand, taurine inhibits the glutamate-induced Ca^2+^ elevation [73]. Another simple explanation for different effects of taurine on ethanol-induced apoptosis in Purkinje and IGL cells may be the difference in time when the neuronal populations were studied. We studied P4 mice at 12 hours and P7 mice at 8 hours after the first ethanol administration. Taurine is readily excreted in urine and its plasma concentration decreases during the experiments. At 8 hours the concentration was still relatively high but at 12 hours almost at the control level; it was not high enough to protect Purkinje cells.

## Conclusions

We show that acute alcohol administration induces apoptosis in Purkinje cells and in cells in the internal granule cell layer (IGL) of the cerebellum. The time frame of sensitivity to ethanol administration is different for Purkinje and IGL cells. Taurine application was neuroprotective against ethanol-induced apoptosis in cells in the IGL. Prevention of caspase-3 activation and DNA fragmentation by taurine in IGL cells is likely to be due to one or several of the following mechanisms, including the restoration of the pool of Bcl-2, regulation of intracellular Ca^2+^ and inhibition of caspase-9 activation. The failure of prevention of ethanol-induced apoptosis by taurine in Purkinje cells may result from the different functional properties of the neurons studied. GABAergic Purkinje cells are inhibitory and glutamatergic granular cells excitatory. The decrease in the taurine level during the experiments may also have an influence since Purkinje cells were studied after a longer interval than IGL cells.

## List of abbreviations used

HPLC: high-performance liquid chromatography; P4: postnatal day 4; P7: postnatal day 7; TUNEL: terminal deoxynucleotidyl transferase-mediated dUTP nick end-labeling; OPA: o-phthaldialdehyde; ANOVA: analysis of variance; IGL: internal granular cell layer; IR: immunoreactive; NMDA: N-methyl-D-aspartate.

## Competing interests

The authors declare that they have no competing interests.

## Authors' contributions

AGT did all laboratory work and drafted the manuscript, EYT performed the statistical analyses and prepared illustrations, IMP participated in invention of the idea and design of the study, MPH was the expert in immunohistochemistry and PS and SSO acted supervisors and composed the final version of this article. All authors read and approved the manuscript.
